# Remediation of Smelter Contaminated Soil by Sequential Washing Using Biosurfactants

**DOI:** 10.3390/ijerph182412875

**Published:** 2021-12-07

**Authors:** Zygmunt Mariusz Gusiatin, Jurate Kumpiene, Ivan Carabante, Maja Radziemska, Martin Brtnicky

**Affiliations:** 1 Department of Environmental Biotechnology, Faculty of Geoengineering, University of Warmia and Mazury in Olsztyn, 10-719 Olsztyn, Poland; 2 Waste Science and Technology, Lulea University of Technology, 97187 Lulea, Sweden; jurate.kumpiene@ltu.se (J.K.); ivan.carabante@ltu.se (I.C.); 3 Institute of Environmental Engineering, Warsaw University of Life Sciences, Nowoursynowska 159, 02-776 Warsaw, Poland; maja_radziemska@sggw.edu.pl; 4 Department of Agrochemistry, Soil Science, Microbiology and Plant Nutrition, Faculty of AgriSciences, Mendel University in Brno, Zemedelska 1, 61300 Brno, Czech Republic; martin.brtnicky@seznam.cz; 5 Institute of Chemistry and Technology of Environmental Protection, Faculty of Chemistry, Brno University of Technology, Purkynova 118, 61200 Brno, Czech Republic

**Keywords:** remediation, soil, copper, lead, saponin, tannic acid, rhamnolipids, organic carbon

## Abstract

This paper presents experimental results from the use of biosurfactants in the remediation of a soil from a smelter in Poland. In the soil, concentrations of Cu (1659.1 mg/kg) and Pb (290.8 mg/kg) exceeded the limit values. Triple batch washing was tested as a soil treatment. Three main variants were used, each starting with a different plant-derived (saponin, S; tannic acid, T) or microbial (rhamnolipids, R) biosurfactant solution in the first washing, followed by 9 different sequences using combinations of the tested biosurfactants (27 in total). The efficiency of the washing was determined based on the concentration of metal removed after each washing (C_R_), the cumulative removal efficiency (E_cumulative_) and metal stability (calculated as the reduced partition index, I_r_, based on the metal fractions from BCR sequential extraction). The type of biosurfactant sequence influenced the C_R_ values. The variants that began with S and R had the highest average E_cumulative_ for Cu and Pb, respectively. The E_cumulative_ value correlated very strongly (r > 0.8) with the stability of the residual metals in the soil. The average E_cumulative_ and stability of Cu were the highest, 87.4% and 0.40, respectively, with the S-S-S, S-S-T, S-S-R and S-R-T sequences. Lead removal and stability were the highest, 64–73% and 0.36–0.41, respectively, with the R-R-R, R-R-S, R-S-R and R-S-S sequences. Although the loss of biosurfactants was below 10% after each washing, sequential washing with biosurfactants enriched the soil with external organic carbon by an average of 27-fold (S-first variant), 24-fold (R first) or 19-fold (T first). With regard to environmental limit values, metal stability and organic carbon resources, sequential washing with different biosurfactants is a beneficial strategy for the remediation of smelter-contaminated soil with given properties.

## 1. Introduction

The continuous increase in pollution of soils, groundwater and sediments constitutes a global environmental threat. High concentrations of copper (Cu), zinc (Zn), lead (Pb), or cadmium (Cd) are detected in ‘hot spots’ in areas associated with mining activities, smelters, metal-containing chemicals, industrial use of waste sludge, fossil fuel combustion, military training, electronics use and waste disposal [1]. Particular remediation challenges are presented in industrial and post-industrial areas, where apart from the presence of elevated concentrations of toxic metals, the soil properties (e.g., low sorption capacity, low organic carbon content, low fertility) are unfavorable for efficient metal stabilization and for plant growth. In the soil environment, heavy metals cannot be degraded or easily removed, and they persist in the soil for a long period of time. Due to high toxicity, heavy metals may show hazardous consequence in the environment and affect human health by biomagnification of the food sources even at low concentrations [1]. The accumulation of high concentrations of toxic heavy metals into soil is harmful to terrestrial flora and fauna, and these toxic metals can also negatively affect soil fertility and crop productivity [2]. Therefore, soils polluted with heavy metals must be remediated to decrease their risks to human health and to improve environmental safety [3].

Several techniques have been proposed over the last years for removal of heavy-metal-contaminated soils in situ (e.g., electrokinetic extraction, soil flushing, phytoremediation) and ex situ (e.g., soil washing) [1]. These techniques differ in efficiency, and the use of many of them is limited due to their high costs [3]. Soil washing can provide long-term benefits by completely remove metals from the soil. This physicochemical method consists of several successive stages: (i) crushing and screening of excavated soil to separate the most highly contaminated fine-soil fractions, (ii) washing of fine soil fractions with a suitable washing solution, (iii) separation of washed soil from the spent washing solution, and (iv) treatment of the spent washing solution and waste disposal [1,4]. For metal removal by soil washing, despite soil properties and metal distribution, the type of washing agent has a crucial effect on the efficiency of soil treatment. Nowadays, the focus in soil washing is gradually shifting from improving extraction efficiency to improving the usability of washed soils, which depends on the type of washing agent.

As a result, the use of biosurfactants, i.e., surfactants of microbial or plant origin, has recently received great interest for environmentally friendly soil remediation strategies. They are becoming increasingly popular as promising agents for remediation (soil washing and soil flushing) and bioremediation of soils contaminated with organic and inorganic pollutants. However, their application has been explored more thoroughly in oil-and hydrocarbon-contaminated soils than in metal-contaminated soils [5]. According to Mulligan [6], biosurfactants should be selected based on pollutant characteristics and properties, treatment capacity, costs, regulatory requirements, and time constraints. Biosurfactants are preferred over synthetic surfactants (e.g., sodium dodecyl sulfate (SDS), Triton X-100, and Tween 80) due to their lower toxicity and higher biodegradability [3,6,7]. Biosurfactants have better foaming properties and higher selectivity. They are active at extreme temperatures, pH (especially plant biosurfactants) and salinity. Microbial biosurfactants (rhamnolipids, sophorolipids, surfactin) can be produced from industrial wastes and byproducts [6]. Unlike microbial biosurfactants, plant biosurfactants (saponins, tannins) can be extracted from various plant parts such as the seeds, fruits, roots, and stems and are often classified as triterpenoids and steroid saponins. This wide distribution could make mass production easier [6]. Depending on the source and purity class, the cost of plant biosurfactant is differentiated, but it is higher than cost of microbial biosurfactants. The main advantages of using biosurfactants derived from plants and microorganisms in soil washing are that they improve the mobility of heavy metal ions by forming micelles, reduce surface and interfacial tension to weaken adhesion between metal ions and the soil, and promote the separation of metal ions from the soil and their complexation with the biosurfactant itself [8]. The use of a biosurfactant solution in soil washing can also increase soil fertility, which improves plant growth [3]. In contrast to conventional washing solutions like inorganic acids, remains of biosurfactants in treated soil can be beneficial because this can lead to soil enrichment with natural organic carbon, which is especially important for soils from industrial areas.

For contaminated soils with aged and multi-metal contamination, the application of a single soil washing might be not enough for an efficient soil treatment. For example, chelating agents have often been reported to be insufficient washing agents when metals are strongly bound to mineral and organic soil constituents [9,10]. Sequential soil washing can facilitate metal removal via selective extraction. Microbial biosurfactants, especially rhamnolipids, are the most popular biosurfactants in various remediation technologies [3]. Besides microbial biosurfactants, plant-derived biosurfactants (e.g., saponin, tannin) are also available on the market. Biosurfactants have different affinities for metals. Rhamnolipids showed higher affinity for Cu and Pb (logK 9.27 and 8.56, respectively) than saponin (logK 6.60 and 3.9, respectively) [11,12]. Tannic acid forms labile complexes with Pb and inert complexes with Cu [13]. Moreover, biosurfactants have different ability to remove metals from individual fractions. Mulligan et al. [14] demonstrated that rhamnolipids and surfactin could remove organically bound Cu, while sophorolipids could remove carbonate- and oxide-bound Zn. Although saponin is effective in metal removal from mobile and stable fractions, tannic acid (3% solution) removed more efficiently Cu, Pb and Zn from the stable fractions in contaminated soil [15]. Hajimohammadi et al. [16] demonstrated that a mixture of saponin and rhamnolipid has a synergistic effect on metal removal from soil. Plant and microbial biosurfactants differ in composition, presence of functional groups, surface-active properties and acidity. The difference in these properties between biosurfactant can differentiate their performance during soil washing and metal removal. Thus, their application in sequential soil washing can be beneficial for simultaneous removal of different metals. All these biosurfactants differ in terms of their synthesis, purification and cost. Thus, using different types of biosurfactants in sequential soil washing, instead of only one, could also decrease the overall cost of remediation [17].

Studies on metal removal via soil washing with biosurfactants have mostly been performed with spiked soils [18,19]. Those studies have focused mainly on optimization of the process in single step washing [20,21], or optionally, on using sequential soil washing with the same type of biosurfactant in each step [19,22,23]. Sequential washing using a combination of different types of biosurfactant in the washing sequence could positively affect metal removal and selected soil properties, particularly stability of residual metals and soil organic carbon. Soil enrichment in organic carbon after soil washing is a result of biosurfactant sorption. The level of biosurfactant sorption depends on its type, concentration, and soil properties. For example, sorption of saponin at its critical micelle concentration (CMC) in soils of different texture (sandy clay loam, clay loam, clay) was 30–53% higher than sorption of rhamnolipids [24].

The aim of this study was to investigate the effect of combined washing with plant-based (saponin, tannic acid) and microbial (rhamnolipids) biosurfactants on Cu and Pb removal from smelter contaminated soil. Due to different properties of plant and microbial biosurfactants, it is important to determine if application of different biosurfactant in washing sequence is more effective for soil remediation than application of one type of biosurfactant in washing sequence and how sequential washing affects metal removal, their stability and biosurfactant sorption in soil. Based on different biosurfactant properties, their affinity for metals and behavior in soil, we hypothesized that the order of biosurfactants used in washing sequence affects Cu and Pb removal, metal distribution and stability in soil as well as biosurfactant sorption in soil. In total, 27 different soil washing sequences were tested in the laboratory. Each variant began with either saponin, tannic acid or rhamnolipids in the first batch of soil washing, followed by nine different combinations of the tested biosurfactants, e.g., saponin-tannic acid-saponin or saponin-saponin-saponin. The objectives of the work were the following: (i) to characterize the initial distribution and stability of metals in soil from the industrial site, (ii) to determine the efficiency of metal removal depending on the application sequence of biosurfactant solutions, (iii) to assess metal distribution and stability in soil after triple washing, (iv) to assess the degree of biosurfactant sorption in soil as a result of the sequential washing, and (v) to assess a relative cost of using biosurfactants in sequential soil washing.

## 2. Materials and Methods

### 2.1. Site Description, Soil Sampling and Characterization

The contaminated site is located in the vicinity of the Legnica Copper Smelter in southeast Poland, which has been in operation since 1953. The current annual production is around 100,000 t of high-quality electrolytic copper, in the form of cathodes and billets. The dust from this smelter is the main source of contamination of surrounding environment.

The soil samples were collected from the topsoil layer (0–30 cm) in deforested terrain at the distance of 150 m from the main emitter. In total, 10 local samples were taken and mixed to obtain a composite sample. The soils were air dried, sieved under 2 mm, homogenized, and characterized in terms of their physicochemical properties. Particle size analysis was determined using a Mastersizer 2000 (Malvern Panalytical Ltd., Malvern, UK). Soil organic carbon was determined by the Tiurin method, based on wet combustion of soil organic matter with potassium dichromate. The soil organic matter was recalculated as organic carbon × 1.724 [25]. The cation exchange capacity (CEC) of the soil was calculated as a sum of the hydrolytic acidity in 1 M Ca(CH_3_COO)_2_ and the exchangeable bases in 0.1 M HCl [25]. The equilibrium pH and electrical conductivity (EC) of the soil in distilled water (1:2.5 ratio, *w/v*) were measured with a pH meter (Hanna HI 221, Hanna Instruments, Woonsocket, RI, USA) and a conductometer (Hanna HI 8733, Hanna Instruments, Woonsocket, RI, USA). The total metal concentration was measured using a flame atomic absorption spectrometer (AA 280FS, Varian, Australia). Before the analysis, the dried soil was digested in aqua reqia (HCl:HNO_3_ at a volume ratio of 3:1) in MARSXpress vessels of 110 mL volume, in a microwave oven (MARSXpress, CEM, Matthews, NC, USA) at 170 °C. The total time of microwave digestion, including reaching and remaining the specific temperature, was 45 min. All soil analyses were performed in triplicates. The main soil characteristics are given in Table 1.

Before sequential soil washing, metal distribution was determined using a modified BCR procedure [26] in which four fractions of different mobility were determined: exchangeable and acid-soluble (mobile F1); reducible (potentially mobile F2); oxidizable (potentially mobile F3); and residual (immobile F4). On the basis of metal distribution, their stability with reduced partition index (I_r_) was determined [18].

**Table 1:** Selected physicochemical characteristics of smelter contaminated soil (mean ± SD, *n* = 3).

Characteristic	Value	
Sand, %	27.3 ± 0.6	
Silt, %	67.9 ± 1.2	
Clay, %	4.8 ± 0.2	
Texture	silt loam	
pH	4.62 ± 0.12	
Electrical conductivity (EC), mS/cm	0.1 ± 0.0	
Soil organic carbon, %	0.47 ± 0.12	
Soil organic matter, %	0.81 ± 0.21	
Cation exchange capacity (CEC), cmol/kg	12.9 ± 0.9	
Total Cu, mg/kg	1651.9 ± 5.6	100/600
Total Pb, mg/kg	290.8 ± 3.5	100/600
Total Zn, mg/kg	165.0 ± 6.1	300/2000

The blue value is the limit metal concentration for agriculture area (group A), the red value is the limit metal concentration for industrial area (group B) according to Polish legislative [27].

### 2.2. Characterization of Biosurfactants as Washing Agents

Three types of biosurfactants were selected for treatment of soil from industrial area: saponin, tannic acid and rhamnolipids. Saponin (product No. 84510) is a secondary plant metabolite being a non-ionic biosurfactant, with a sapogenin content of 8–25%. Tannic acid (product No. 16201) is a naturally occurring plant polyphenol belonging to hydrolyzable tannin. The biosurfactant (C_76_H_52_O_46_, 1701.2 g/mol) consists mainly of gallic acid residues linked to glucose via glycosidic bonds. Both plant biosurfactants are in the form of powder and were purchased from Sigma-Aldrich (Burlington, MA, USA). Rhamnolipids are a 25% mixture of anionic rhamnolipids of type R1 (C_26_H_48_O_9_) and type R2 (C_32_H_58_O_13_) with trade name of JBR425. The biosurfactant is produced by *Pseudomonas aeruginosa* from fermentation broth resulting in a dark brown and viscous liquid which was purchased from Jeneil Biosurfactant Co LLC (Saukvile, WI, USA). All three biosurfactants were used without further purification.

In this study, it was assumed that the concentrations of all washing solutions were the same. The concentration was selected on the basis of total organic carbon (TOC) concentration in 3% saponin solution [18,28]. To prepare 3% solution of saponin, 30.0 g of biosurfactant powder was dissolved in 1 L of distilled water using a magnetic stirrer, which corresponded to TOC concentration of 13.6 g TOC/L. To prepare tannic acid and rhamnolipids solutions at comparable TOC concentration as saponin, 25.4 g of tannic acid and 90.3 g of rhamnolipids were dissolved in 1 L of distilled water. In biosurfactant solutions, the following characteristics were measured: pH, EC, TOC with Shimadzu Liquid TOC-VCSN analyzer (Shimadzu Corporation, Kyoto, Japan) and surface tension with Krüss K100 tensiometer (Krüss GmbH, Hamburg, Germany). The TOC in biosurfactant solutions was calculated by subtracting the inorganic carbon from the total carbon. The basic characteristics of each biosurfactant solution are shown in Table 2.

### 2.3. Sequential Batch Soil Washing

**Figure 1:**
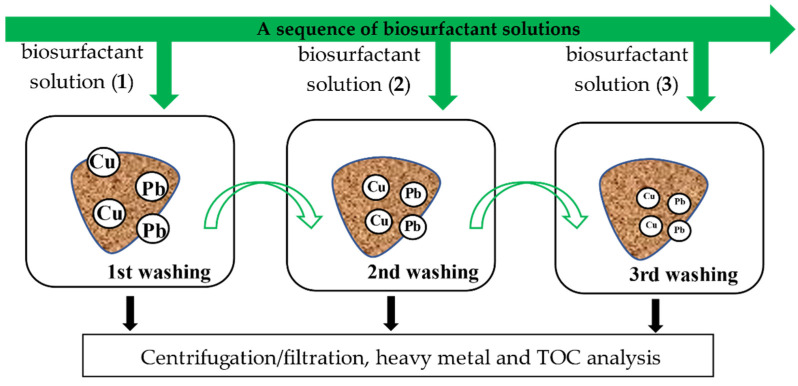
Sequential soil washing procedure with biosurfactant solutions.

All the soil washing experiments were conducted in 50 mL polyethylene tubes with a soil to biosurfactant solution ratio of m/V = 1/40 (*w/v*). A series of sequential batch washings was performed in two replications. Three main types of washing variants, based on the type of biosurfactant used in the first soil washing, were tested: saponin (S) variant, tannic acid (T) variant and rhamnolipids (R) variant. In each variant, nine different combinations of the tested biosurfactants were used (Table 3, Figure 1).

These sequences differed in the type of biosurfactant used in an individual soil washing (glycoside, polyphenol or glycolipid) and in the order of the individual biosurfactants used in the sequential soil washing. In total, 27 soil washing sequences were tested. The samples were shaken in an Intelli Mixer RM-2L at 90 rpm for 2 h for single washing at room temperature (22–24 °C).

At the end of each washing, the samples were centrifuged at 8000 rpm for 20 min and filtered through 0.45 µm filters under vacuum. The concentration of heavy metals and TOC were measured in the supernatants. To assess cumulative effect on metal removal and organic carbon balance, the soil residue was not washed with distilled water between each soil washing step.

### 2.4. Data Elaboration and Statistical Analysis

Total metal concentration in contaminated soil (C_M_) and the removed metal concentration from soil during soil washing (C_R_) was calculated according to Equation (1):

(1) $$C_{M\;}{\mathrm{or}\;C}_{R}=\frac{(C_{s/r}\times f)-C_{c}}{m}\times V$$

where C_S_ is metal concentration in soil extract after microwave mineralization, C_r_ is metal concentration in supernatant after soil washing (mg/L), C_c_ is metal concentration in blank sample (mg/L), f is dilution factor, V is the total volume of liquid sample after microwave mineralization or the volume of supernatant after soil washing (L), m is soil weight (kg of dry mass).

Metal stability in contaminated and washed soil, based on their chemical fractionation, was calculated according to Equation (2):

(2) $$I_{r}=\overset{k}{\underset{i=1}{\sum }}i^{2}\times F_{i}/k^{2}$$

where i is the index number of the BCR sequential extraction step, progressing from 1 (for F1 fraction) to 4 (for F4 fraction), k is the total number of fractions in the BCR procedure, F_i_ is relative metal content in fraction i. The classification of metal stability based on the I_r_ is as follows: lack of stability (I_r_ ≤ 0.1), low stability (0.1 < I_r_ ≤ 0.3), medium stability (0.3 < I_r_ ≤ 0.5), elevated stability (0.5 < I_r_ ≤ 0.7), high stability (0.7< I_r_ ≤ 0.9) [29].

The cumulative efficiency of metal removal with biosurfactants during three-consecutive soil washing (E_cumulative_) was calculated according to Equation (3):

(3) $$E_{\mathrm{cumulative}}=\frac{\sum {\left(C_{R}\right)}_{n}}{C_{M}}\times 100$$

where n is the number of soil washing with a given sequence of biosurfactant solutions.

For data with significant differences identified by ANOVA, further analyses were conducted using Tukey’s HSD test (Statistica 13.1, TIBCO Software Inc., Palo Alto, CA, USA). A Pearson correlation coefficient was calculated to estimate the relationship between E_cumulative_ and the I_r_ for Cu and Pb removed with different biosurfactant variants (*p* < 0.05, Statistica 13.1, TIBCO Software Inc, Palo Alto, CA, USA). The value for a Pearson’s correlation is between 0.00 (no correlation) and 1.00 (perfect correlation). A correlation >0.80 is considered strong, and <0.50 is weak.

## 3. Results and Discussion

### 3.1. Metal Distribution and Stability in Smelter Contaminated Soil

**Figure 2:**
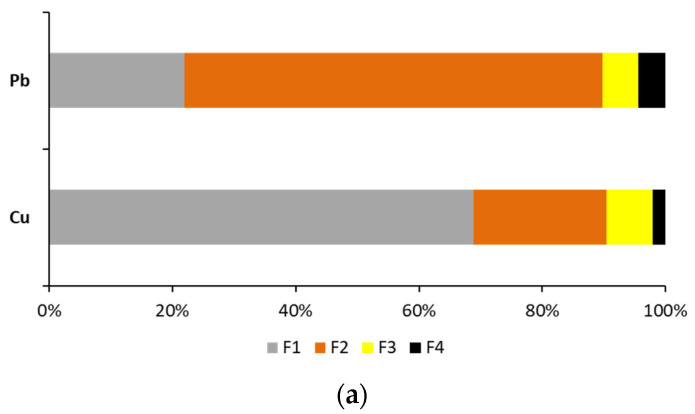

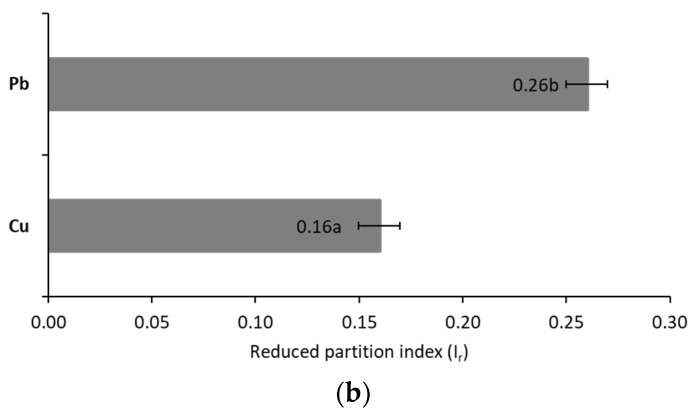
Metal distribution (**a**) and stability (as I_r_) (**b**) in smelter contaminated soil. For I_r_, different letters indicate significant differences in metal stability in soil (*p* < 0.05).

One of the factors that influences metal removal by soil washing is the distribution of metals in the soil, i.e., their presence in particular chemical forms (fractions). In the soil from industrial areas, the analyzed metals differed in total concentration and distribution pattern (Figure 2).

Metals in the exchangeable and acid soluble fraction (F1) are considered to be the most mobile in the soil. This is because they are present in ionic form, bound to carbonates, and exchangeable, due to electrostatic adsorption [30]. In addition, metals in the F1 fraction are susceptible to changes in pH. In the soil from industrial areas, the fractionation of Cu (69%) in the F1 fraction was high, whereas that of Pb was lower (22%). Lead had a larger share in the reducible fraction (68%) than Cu (22%). Metals can form complexes with functional groups on oxide surfaces and be (co)precipitated or strongly bonded [31]. Metal oxides, hydroxides and oxyhydroxides, due to their high surface reactivity and large surface areas, play an important role in heavy metal sequestration in soil, especially with regard to Pb [32]. Pb demonstrates a strong affinity for adsorption on Fe, Mn and Al oxides, and it is co-precipitated with Fe and Mn oxides [33].

Depending on the oxide form (easily reducible Mn oxides, moderately reducible amorphous Fe oxides, poorly-reducible crystalline Fe oxides), the mobility of metals in this fraction can vary. Metals associated with Mn oxides are more mobile than those associated with amorphous Fe oxides [34]. The reducible fraction that is extracted with the BCR protocol that was used in this study includes metals bound to amorphous Fe and Mn oxides and hydroxides [35]. Usually, Cu and Pb display a strong affinity for soil organic matter, and the metals can be immobilized via specific adsorption reactions [33]. Due to the low organic matter content in the soil from industrial area, the shares of the metals in the oxidizable fraction were low, 5.8% for Pb to 7.4% for Cu (Figure 2a). In the most stable F4 fraction, Cu and Pb had, respectively, only 2% and 4% of their total concentration. The relatively small proportions of the metals that were present in the F4 fraction can be attributed to the low clay content of the soil from industrial area [36].

The distribution patterns of Cu and Pb in the present study are similar to those in soil samples collected close to (70 m) the Legnica Cu smelter [37]. Although the total metal concentrations were markedly higher in that study, Cu was present mainly in the exchangeable and acid soluble fraction (73.1%), while Pb was present mostly in the reducible fraction (64.9%).

Based on metal fractionation and the I_r_, the stability of Cu and Pb was low (Figure 2b). Similarly, in the soil samples collected 70 m from the Legnica Cu smelter, all the analyzed metals had low stability, which decreased in this order: Pb (0.20) > Cu (0.15) [37]. The source of contamination affects the mobility and stability of metals in soil. For example, in soil from a steel disposal dump, Cu had medium stability (0.44), while Pb had low stability (0.24) [38]. According to Lopes et al. [39], soil from a smelting site contains metals in a more mobile and bioavailable form, which potentially might cause more severe environmental problems than those in a mining area. On the basis of metal fractionation and stability, the soil from the industrial area in the present study demonstrated potential to be effectively treated with soil washing. During sequential washing, metals can be removed from the most mobile fraction (e.g., exchangeable) first, and then from the less mobile fractions (e.g., reducible) [14]. In addition, the soil showed low organic matter content and low CEC. Soils with less than 10–20% clay and low organic content (a CEC of between 0.5 and 10 cmol/kg) can be most effectively cleaned by soil washing [14].

### 3.2. Effect of Sequential Soil Washing with Biosurfactants on Metal Removal from Smelter Contaminated Soil

**Figure 3:**
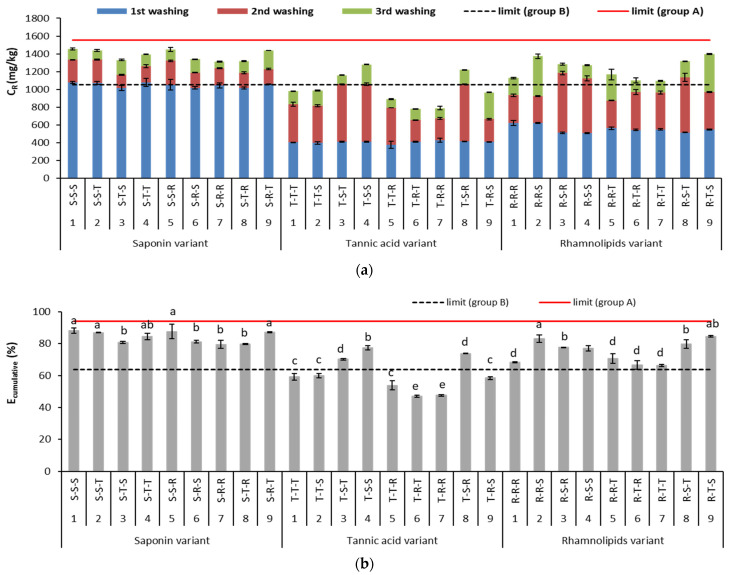
Concentration of metal removed (C_R_) (**a**) and cumulative efficiency of Cu removal (E_cumulative_) (**b**) during sequential soil washing. For E_cumulative_, different letters indicate significant differences in metal removal efficiency with different biosurfactant sequences (*p* < 0.05). Limit (group A) and limit (group B) mean minimum Cu concentration to be removed from soil or minimum Cu removal efficiency to meet limit values for agricultural and industrial areas, respectively.

**Figure 4:**
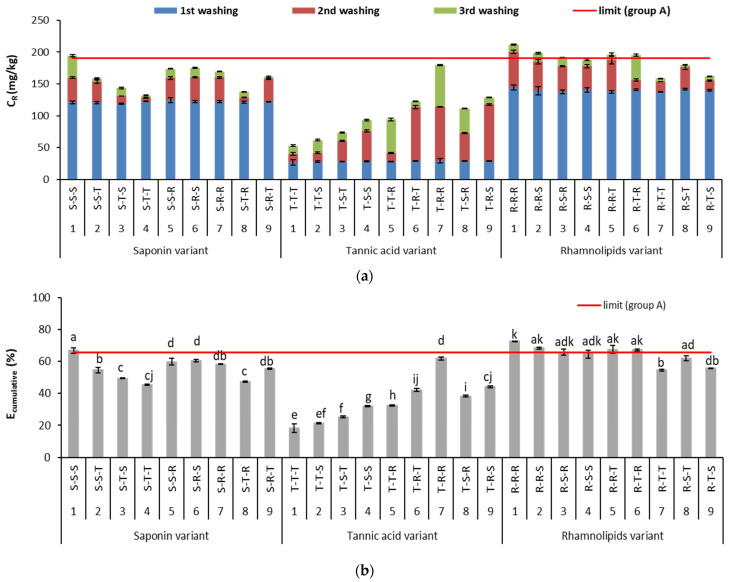
Concentration of metal removed (C_R_) (**a**) and cumulative efficiency of Pb removal (E_cumulative_) (**b**) during sequential soil washing. For E_cumulative_, different letters indicate significant differences in metal removal efficiency with different biosurfactant sequences (*p* < 0.05). Limit (group A) means minimum Pb concentration to be removed from soil or minimum Pb removal efficiency to meet limit value for agricultural area.

In the contaminated soil tested in this study, Cu and Pb, in contrast to Zn, exceeded limit concentrations in soil according to Polish legislation (see Table 1). Therefore, soil washing with biosurfactants was used for the removal of only these two metals. The effect of sequential washing on soil treatment was assessed on the basis of the concentrations of metal removed (C_R_) after individual washings and on the cumulative removal efficiency (E_cumulative_). Both indicators changed depending on the main variant (i.e., which biosurfactant was used first) and on the types of biosurfactants used in the subsequent steps of the soil washing. The results of metal removal in consecutive washings and the overall efficiency of metal removal are presented in Figure 3 and Figure 4.

In the variant in which saponin was used first in the sequence of washings, Cu removal was the highest (1047 mg/kg, on average) (Figure 3a). With each successive washing, C_R_ decreased, and after the third washing, it was only 4.6–12.8 mg/kg. The E_cumulative_ of Cu in the saponin-first variant ranged from 47 to 88%. Four washing sequences gave the best results: S-S-S, S-S-T, S-S-R and S-R-T (1444 mg/kg and 87.4%, on average).

Cu removal after the first washing was lower in the variants in which tannic acid and rhamnolipids were used first than in the saponin-first variant, averaging 405 mg/kg for tannic acid and 552 mg/kg for rhamnolipids in the first washing (Figure 3a). In the second and third stages of the tannic-acid-first and the rhamnolipid-first variants, Cu removal was most efficient when saponin was used. The E_cumulative_ of Cu in the tannic-acid-first variant ranged from 47 to 77%, with the highest efficiency in the T-S-S and T-S-R sequences. In the rhamnolipid-first variant, the E_cumulative_ ranged from 67 to 84%, and Cu removal was highest in the R-T-S and R-R-S sequences (Figure 3b).

In the present study, all biosurfactants were used at concentrations above their CMC, which means micelle formation and complexation of metals with functional groups were responsible for metal removal. Saponin contains 10-times more carboxylic groups on its hydrophilic head than tannic acid. These groups have a greater tendency to deprotonate under acidic conditions than phenolic groups, which facilitates simultaneous removal of different metals, including Cu, Pb and Zn [15,40]. However, the affinity of saponin for metals can differ. Based on the stability constants (logK) for metal complexes with saponin in aqueous solution, the biosurfactant forms complexes with Cu to a greater extent than it does with Pb: logK Cu (6.60) > logK Zn (4.32) > logK Cd (4.12) > logK Pb (3.9) [12,41]. Hong et al. [28] showed that saponin was very effective for Cu removal from soils with low organics content. Those authors reported that, after a single washing of sandy clay loam containing 0.07% organic matter, Cu removal was 62%. With soils containing more organic matter (7.2–11.2%), the removal of Cu with saponin was markedly lower due to stronger bonding of Cu and sorption of saponin in the soil. Similarly, it was reported that, with light soil, 82% of Cu was removed after the first washing, whereas with heavy soil, Cu removal increased significantly after each step, for a cumulative removal efficiency of 65% after triple washing [18]. Thus, sequential soil washing with saponin is a better option for heavier soils, e.g., those with a silty clay texture, rather than lighter soils, e.g., those with a loamy sand texture.

Sequential soil washing with the use of different biosurfactants in each washing step can increase the efficiency of removal of different heavy metals due to complementary complexation caused by differences in the affinity between the heavy metals and the biosurfactants [42]. On the other hand, during each washing step, a new equilibrium distribution of residual metals is reached [43], and individual biosurfactants can be retained in soil to various degrees due to their sorption. This can result in changes in soil chemistry, which might affect both metal–biosurfactant and biosurfactant–biosurfactant interactions in subsequent washings. Hajimohammadi et al. [16] found that mixed rhamnolipids and saponin had a synergistic effect on heavy metal removal from an oil contaminated soil. Biosurfactants used in such a configuration were able to create a film around the dispersed phase and improve the reinforced interfacial film, consequently increasing the rate of micelle formation responsible for metal removal. In this study, the efficiency of the biosurfactant variants for Cu removal decreased in this order: saponin > rhamnolipids > tannic acid (Figure 3b). Sequential soil washing for Cu removal could be more reasonable for the variants that used tannic acid or rhamnolipids first. When only a single soil washing was applied, saponin removed most of Cu. All biosurfactant sequences in the saponin-first and rhamnolipid-first variants met the requirements for industrial areas (min. 63.7% Cu removal efficiency, i.e., 1052 mg/kg [27]), but only three sequences from the tannic-acid-first variant met the requirements (T-S-T, T-S-S, T-S-R). None of the tested variants met the limit values for agricultural areas (93.9%, i.e., 1552 mg/kg [27]).

The type of biosurfactant that was used strongly affected Pb removal. When saponin or rhamnolipids were used first, Pb removal in the first washing averaged 122 mg/kg or 140 mg/kg, respectively. In contrast, when tannic acid was used first, it averaged only 28 mg/kg. Among the tested biosurfactants, rhamnolipids had the strongest affinity for Pb.

Saponin removed less Pb than rhamnolipids. This is because, in the soil, the Cu concentration was higher than that of Pb, and there was strong competition between Cu and Pb for the carboxylic groups in saponin [15]. As for tannic acid, it is a polyphenol, i.e., an organic ligand with a high molecular weight and numerous phenolic groups that can form complexes with metals [42,44]. In soil, tannic acid can react with Fe, decomposing Fe oxides and facilitating release of metals bound to oxides [22]. Nevertheless, in this study, tannic acid removed relatively little Pb from soil. This could be because all biosurfactants were used at the same TOC-based concentration (ca. 13 g/L). However, the optimum concentration of tannic acid for simultaneous removal of multi-metals from soil is ≥20 g TOC/L [20]. At higher concentrations, tannic acid is more acidic due to the presence of more acidic phenolic groups, which facilitate removal of metals like Pb [15,42].

In all biosurfactant variants, a second washing with saponin or rhamnolipids removed more Pb than one with tannic acid. The highest increase in the C_R_ for Pb (86 mg/kg, on average) was obtained after the second washing with rhamnolipids in the tannic acid variant. After sequential soil washing, the E_cumulative_ was 45–67% in the saponin-first variant, 18–62% in the tannic-acid-first variant and 54–68% in the rhamnolipids-first variant. The total Pb concentration in the soil met the requirements for areas from group B; thus, the results were only compared with the limit for areas from group A (100 mg/kg). In order to meet this limit, the min. C_R_ for Pb in this soil should be 191 mg/kg, and the minimum removal efficiency should be 65.6% [27]. This requirement was met by most sequences in the rhamnolipids-first variant that used a rhamnolipids solution two or three times. In the saponin-first variant, only triple washing with saponin met the requirement. In contrast, none of the sequences from the tannic-acid-first variant met the requirement (Figure 4b).

Rhamnolipids have a higher affinity for transition heavy metals, including Cu and Pb, than for alkaline metals [45], as shown by the stability constants for the rhamnolipids-metal chelates (log K): Al > Cu > Pb > Cd > Zn > Fe > Ca > Co > Ni > Mn > Mg > K [46]. These biosurfactants possess one or two rhamnosil groups and carboxyl groups [47], and they have an anionic character at a pH of 6.8 [48]. Rhamnolipids are easily deprotonated, and they can decrease surface and interfacial tension, which favours interaction with cationic metals and formation of organic salts [49]. In this study, the rhamnolipids solution had the highest pH (6.4, Table 2). At pH > 6.0, rhamnolipids are predominately small vesicles and micelles [50], which can facilitate movement of biosurfactant-metal complexes. Rhamnolipids (e.g., the R-R-R sequence) removed more Pb than saponin (e.g., the S-S-S sequence) because the stability constant (log *K*) of Pb-rhamnolipids chelates an in aqueous solution is more than two times higher than that of Pb-saponin chelates [11,51].

The results on Pb removal that are presented here agree with those of previous studies on sequential soil washing with different biosurfactants. Double washing with rhamnolipids removed Pb with a cumulative efficiency of 68–73% from two spiked soils differing in properties and the length of soil aging and containing multi-metals at various concentrations. Double washing with a sequence of saponin followed by rhamnolipids also effectively removed Pb (50–56%) [42].

### 3.3. Effect of Sequential Soil Washing with Biosurfactants on Metal Distribution and Stability

**Figure 5:**
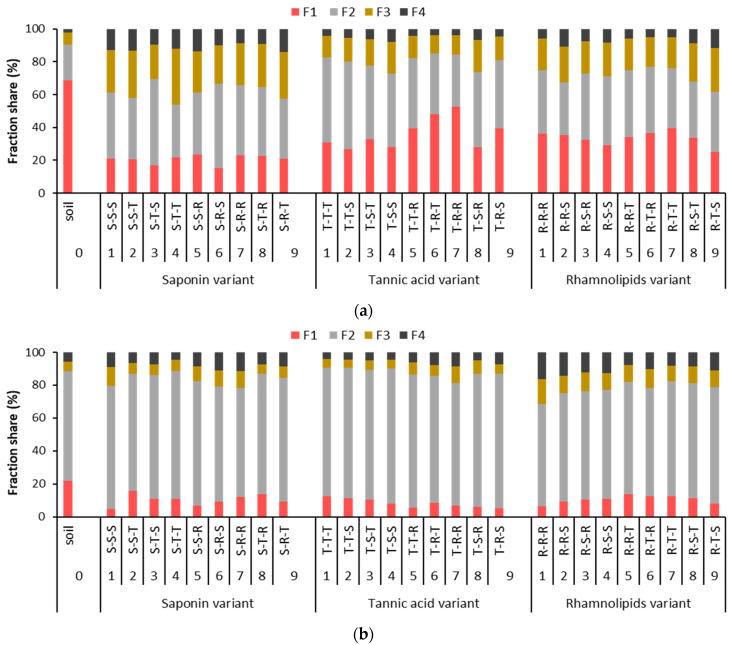
Changes in the share of individual metal fractions in soil after triple washing with the use of saponin, tannic acid or rhamnolipids variants: (**a**) Cu, (**b**) Pb (soil 0 refers to unwashed soil).

The performance of individual biosurfactants in sequential soil washing depends on the metal distribution in soil. Biosurfactants can change metal fraction distribution so that metal stability in soil is higher after washing than before washing [42]. To considerably increase metal stability, their mobile fractions should be removed as much as possible. Poor metal removal from stable fractions (F3 and F4) or even an increase in metal shares in these fractions can increase metal stability. The changes in the shares of individual fractions of Cu and Pb in soil after triple washing are presented in Figure 5.

Most of the Cu in the contaminated soil was in the exchangeable and acid soluble fraction (F1, 69%). Thus, removing or redistributing the Cu in this fraction had the largest effect on Cu removal and the stability of the Cu remaining in the soil. In general, triple washing decreased the share of Cu in the F1 fraction and increased its share in the F2, F3 and F4 fractions. The saponin-first variant decreased Cu mobility most effectively, and the R-T-T sequence decreased it least effectively. The saponin-first variant changed the share of Cu in the F1 fraction to a smaller extent than the rhamnolipids-first and the tannic-acid-first variants (15.3–23.6%, 25.2–39.4%, 24.3–52.8%, respectively).

Although Cu was removed from the F2 fraction, the share of metal remaining in this fraction fluctuated depending on the washing sequence, and an overall trend was not obvious. The shares of Cu in the F2 fraction and the ratio of the residual Cu concentration in the F2 fraction to that in the F1 fraction were highest after washing with these sequences: T-T-T (52.0%), T-T-S (53.4%), S-T-S (52.6%) and S-R-S (51.1%) (Figure 5a). In contrast, the shares of Cu in the F2 fraction were lowest after these sequences: R-R-S, S-T-T and T-S-R. The residual Cu concentration in the F2 fraction averaged 114.5 mg/kg (saponin-first variant), 263.6 mg/kg (tannic-acid-first variant) and 165.1 mg/kg (rhamnolipids variant).

Saponin is known to efficiently remove Cu removal from the F1 and F2 fractions; if the metals are predominantly in these fractions and the soil properties are suitable, most Cu can be removed already after single washing. For example, in loamy sand and loam soils containing on average 1171 mg/kg of Cu, a single washing with saponin removed over 80% of Cu from the F1 fraction and 44–54% from the F2 fraction [18]. Its strong affinity for metals enables this biosurfactant to efficiently remove metal from not only soil, but also municipal sewage sludge [41] or organic fertilizers like pig manure [52]. Hong et al. [28] have shown that in soil highly contaminated with heavy metals, including Cu (1521–2181 mg/kg) and Pb (5253–7288 mg/kg), saponin was able to mobilize metals from the reducible (F2) and oxidizable (F3) fractions, but removed more metal from the exchangeable and carbonate fractions (=F1 fraction in the present study). Tannic acid can remove metals from mobile and immobile fractions (i.e., reducible, organic and residual) more efficiently than saponin when used at a higher concentration and lower pH [15].

The share of the most stable F3 and F4 fractions in washed soil was highest after application of sequences from the saponin variant, and the lowest after application of sequences from the tannic acid variant. This is because Cu concentration in F1 and F2 fractions in soil washed with the use of tannic acid variant was still high.

Sequential soil washing decreased Pb mobility in soil. The type of washing sequence was important in decreasing Pb mobility as was indicated by some dynamic changes in the share of F1 fraction. In general, a lower share of Pb in the F1 fraction was found in soil washed in tannic acid variant than in saponin or rhamnolipids variants (Figure 5b). In soil from industrial area, Pb prevailed in reducible (F2) fraction (Figure 2a). Using saponin variant, the removal of Pb from F2 fraction varied from 36.3% (S-T-T) to 57.5–59.6% (S-R-R, S-R-S). With the tannic acid variant, Pb was removed less efficiently from the F2 fraction. The highest removal (54.4%) was obtained for T-R-R sequence. Thus, in soil washed with tannic acid variant, Pb share in the F2 fraction was the highest. In rhamnolipids variant, Pb was removed from the F2 fraction with the efficiency, 50.3–74.5%.

The highest removal was obtained for R-R-R sequence. Sequential soil washing with rhamnolipids and saponin variants was more beneficial to increase Pb share in F3 and F4 fractions as compared to the original contaminated soil. In the case of F4 fraction, the highest shares (13–14%) were obtained for sequences where only rhamnolipids were used, or rhamnolipids with saponin.

In our previous studies, saponin, tannic acid or rhamnolipids used in single washing of two different soils (soil 1 and 2) spiked with mixed metals effectively removed Pb from the F1 fraction. These biosurfactants differed visibly in Pb removal from the F2 and F3 fractions. Rhamnolipids decreased Pb concentration in potentially mobile fraction (sum of F2 and F3) by 55–59%, while saponin and tannic acid by 5–13% (soil 1) and 23–26% (soil 2) [42].

### 3.4. A Relationship between Cumulative Metal Removal Efficiency and Their Stability

**Figure 6:**
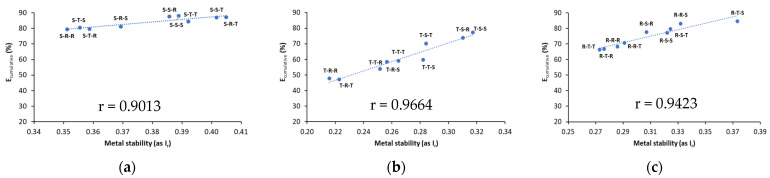
A relationship between cumulative Cu removal efficiency (E_cumulative_) and stability of residual Cu in washed soil (I_r_) for different biosurfactant variants: (**a**) saponin variant, (**b**) tannic acid variant, (**c**) rhamnolipids variant.

**Figure 7:**
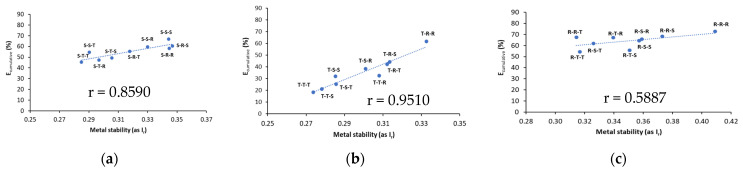
A relationship between cumulative Pb removal efficiency (E_cumulative_) and stability of residual Pb in washed soil (I_r_) for different biosurfactant variants: (**a**) saponin variant, (**b**) tannic acid variant, (**c**) rhamnolipids variant.

To analyze how metal removal with each tested sequence from a given washing variant correlates with metal stability in soil, a relationship between E_cumulative_ and reduced partition index (I_r_) was evaluated. The results are shown in Figure 6 and Figure 7.

With all biosurfactant variants, Cu and Pb stability (as I_r_) in washed soil increased considerably as a result of changes in metal distribution patterns (Figure 6 and Figure 7). For most cases, a very strong correlation (r > 0.8) was demonstrated between E_cumulative_ and the stability of residual metals in soil (I_r_).

Cu stability in washed soil was 0.35–0.40 for saponin variant, 0.22–0.32 for tannic acid variant, and 0.27–0.37 for rhamnolipids variant. Taking into consideration the highest removal efficiency of Cu and the highest Cu stability in each variant, the most appropriate washing sequences were S-S-T, S-R-T, T-S-S and R-T-S (Figure 6). With these sequences, the average removal efficiency of Cu was 87.4%, and the average Cu stability was 0.40 (medium stability). Among all variants, application of sequences from saponin variants resulted in higher Cu stability compared to the sequences from tannic acid and rhamnolipids variants. This was related to more efficient decrease in Cu mobility, i.e., the content of F1 fraction in saponin variant. Gusiatin et al. [42] also obtained higher Cu stability in different soils using single washing with saponin, compared to single soil washing with tannic acid or rhamnolipids.

For Pb, its stability in soil washed with saponin variant was 0.28–0.34, with tannic acid variant, 0.27–0.33, and with rhamnolipids variant, 0.32–0.41. The most suitable sequences for Pb removal and its stability using individual variants were S-R-S, S-R-R, S-S-S, T-R-R and R-R-R (Figure 7). However, the highest Pb removal (64–73%) and the highest Pb stability (0.36–0.41) was observed for four sequences of the rhamnolipids variant: R-R-R, R-R-S, R-S-R and R-S-S (Figure 7c).

In recent studies on soil washing, the I_r_ was a useful tool to evaluate metal-binding intensity in different matrices (soil, sludge), contaminated with different metals at different concentrations, washed under different conditions with various washing agents and with different effectiveness of metals removal [29]. Tang et al. [51] demonstrated that after triple washing of sewage sludge with combined rhamnolipid and saponin, the stability of Cu increased from 0.23 to 0.47, and that of Pb from 0.25 to 0.55. This is because metals were easily removed from exchangeable (F1) and reducible (F2) fractions, while some portion of oxidizable (F3) and residual (F4) fractions were still difficult to remove.

### 3.5. Effect of Sequential Soil Washing on Biosurfactant Sorption in Soil

**Figure 8:**
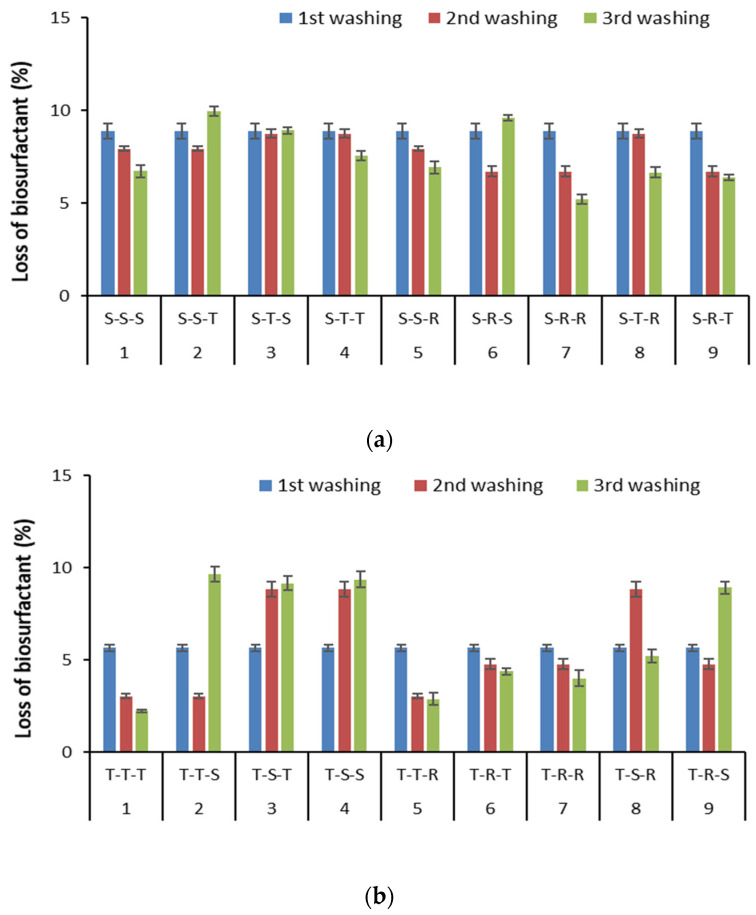

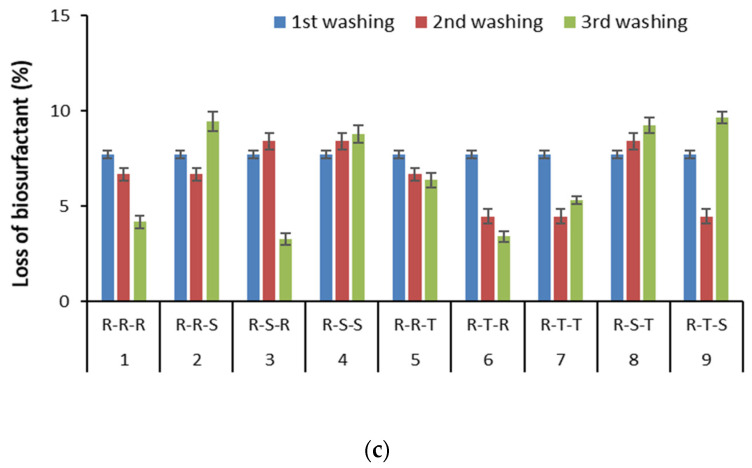
Estimation of biosurfactant loss after individual soil washing using specific biosurfactant sequence: (**a**) saponin variant, (**b**) tannic acid variant, (**c**) rhamnolipids variant (mean ± SD, *n* = 2).

Based on the comparison of TOC concentration in biosurfactant solutions before and after sequential soil washing, their loss due to sorption in soil was possible to be estimated (Figure 8).

It was observed that in each step of the sequential soil washing, the loss of biosurfactants did not exceed 10%. A relatively low loss of biosurfactant can be associated with the soil composition. High organic matter and clay content in soil facilitate biosurfactant sorption into soil [24,53]. In this study, the soil showed low content of organic matter and clay. Using sequential washing with the same type of biosurfactant, the highest loss was after the first washing, and it decreased gradually in subsequent washings (Figure 8). When sequences of mixed biosurfactants were used, the level of their loss depended on the type of biosurfactant used in the previous washing. In general, saponin or tannic acid used after each other did lower their sorption. Rhamnolipids used after saponin or tannic acid showed lower loss, while saponin used after rhamnolipids showed greater loss. An opposite trend was observed for tannic acid used after rhamnolipids. These results indicate that the interaction of the biosurfactant with each other ultimately affects their retention in soil. Different mechanisms are involved in biosurfactant sorption in soil, such as hydrogen bonding, electrostatic attraction, and hydrophobic interactions [54,55].

**Figure 9:**
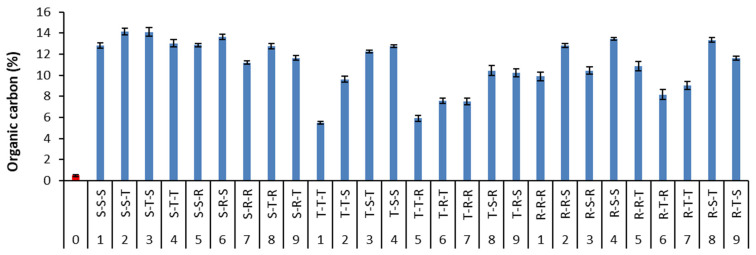
Enrichment of smelter contaminated soil with organic carbon as a result of sequential soil washing with biosurfactants (mean ± SD, *n* = 2).

As a result of biosurfactant loss, cumulative enrichment of soil with organic carbon was calculated and compared with organic carbon content in the contaminated soil before washing (Figure 9). Sequential soil washing with biosurfactants was beneficial, increasing organic carbon resources in remediated soil. Soil organic carbon is the most important component for maintaining soil quality (physical, chemical and biological soil properties) and improving many soil functions [56].

In the present study, the type of biosurfactant sequence played a role in the level of organic carbon in soil. Organic carbon accounted for only 0.47% before washing, whereas it was increased to an average of 12.9% (saponin variant), 9.1% (tannic acid variant) and 11.1% (rhamnolipids variant) after sequential washing. However, the stability of external organic carbon in biosurfactant-washed soil needs further investigations. Our previous work demonstrated that tannic acid used for flushing metals from contaminated soil increased soil organic matter from 4.1% (unflushed soil) to 10.4–10.8% (flushed soil) [57]. Tannins can take part in the formation of humic-like substances because reactive quinones from tannins can self-polymerize or co-polymerize with other compounds such as amino-containing compounds [58]. Thus, biosurfactants of larger molecular structure, such as saponin and tannic acid, retained in soil can provide some portion of stable carbon in remediated soil. Further investigations are necessary on the stability of external organic carbon in soils remediated with biosurfactant washing.

### 3.6. Assessment of Relative Cost of Using Biosurfactants in Sequential Soil Washing

**Figure 10:**
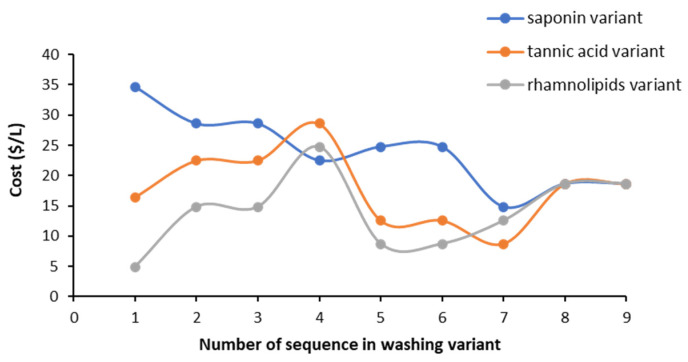
A cumulative relative cost of biosurfactant solutions used in triple washing with washing sequences from saponin, tannic acid and rhamnolipids variants (cost for 1 L of biosurfactant solution used in each washing step, m/V ratio 1/40).

To evaluate which of the tested washing sequences was the most cost-effective, a relative cost was evaluated based on the cost of biosurfactant solutions and number of washings. In the cost assessment (Figure 10), the price of biosurfactants was taken from Sigma-Aldrich (saponin $850/kg, tannic acid $215/kg, 2021) and Jeneil Biosurfactant Co. LLC, Saukville, WI, USA) (rhamnolipids $18/kg, 2020) ($ = USD).

The data presented in Figure 10 indicate that triple soil washing using only saponin is the most expensive ($34.7/L), while triple soil washing with only rhamnolipids is the cheapest ($4.9/L). The use of two or three types of biosurfactants in the washing sequence could lead to a more cost-effective treatment, especially for saponin variant (e.g., sequences 4, 7) and for tannic acid variant (sequences 5–7).

**Figure 11:**
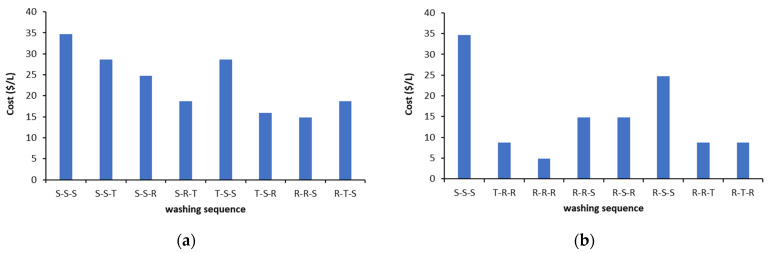
Comparison of relative cost for the most effective sequences for treatment of smelter contaminated soil selected on the basis of metal removal, metal stability and organic carbon enrichment: (**a**) Cu, (**b**) Pb.

Taking into consideration Cu and Pb removal efficiency, metal stability and organic carbon content in remediated soil, the most effective washing sequences were selected among the 27 different treatments (Figure 11). The most cost-effective sequences giving similar results in terms of soil remediation were T-S-R, R-R-S for Cu and R-R-R for Pb.

Application of biosurfactants, beside their environmental advantages, is relatively expensive. The development of cheaper production routes for biosurfactants, for instance finding new and cheaper sources, is crucial for a wider application of these solvents in soil washing practices. The current market prices of biosurfactants varies depending on the level of purity and manufacturer [59]. For example, NutraHerb Co., Ltd. (Xi’an, Shaanxi, China), sells high quality *Quillaja saponaria* bark extract in the form of powder rich in saponin, at a price of $10–30/kg (2021). Haihang Industry Co., Ltd. (Jinan City, China) sells tannic acid of food grade at a price of $100/kg (2021). The cost of biosurfactant is also inversely proportional to the scale of their production, e.g., production cost is $20/kg in a batch of 20 m^3^, whereas the cost reduces to $5/kg in a batch of 100 m^3^ [60]. Commercial plant biosurfactants after soil washing could be recovered by separation of pollutants from the effluent prior to reutilization. The recovery and reuse of biosurfactants make soil washing more environmentally friendly and cost-effective [61]. Thus, in practice, the cost of using biosurfactants in soil remediation could be much lower than the relative cost indicated in the present study. Nevertheless, the use of different types of biosurfactants in sequential soil washing could decrease remediation cost and still achieve relatively high washing performance.

## 4. Conclusions

Remediation of smelter contaminated soil was performed, employing sequential washing starting with saponin (nine sequences), tannic acid (nine sequences) or rhamnolipids (nine sequences). The concentrations of Cu and Pb removed after individual washings and the cumulative efficiencies of removal of these metals depended on which biosurfactant was used first as well as the types of biosurfactants used in the washing sequence. In contaminated soil, Cu and Pb differed mostly in the share of exchangeable and reducible fractions. The sequential washing had a substantial effect on the change in Cu and Pb distribution patterns and their stability. As the cumulative efficiency of Cu and Pb removal increased, so did the stability of the metal that remained in the soil, from low (contaminated soil) to medium level (washed soil). Cu removal and stability were the highest after washing with sequences that started with saponin or that finished with saponin after, either a double washing with rhamnolipids or a single washing with rhamnolipids and a second washing with tannic acid. Pb removal was the highest after sequences containing only rhamnolipids or combinations of rhamnolipids and saponin. Sequential washing also substantially increased organic carbon content in the soil due to biosurfactant sorption (23 times higher after washing, on average).

The overall results indicate that sequential washing with biosurfactants showed promising results in the treatment of real metal-contaminated soil. The wide range of tested washing sequences makes it possible to choose the most appropriate option in terms of the cost and the purpose of remediation.

The study provides knowledge about performance of plant and microbial biosurfactants used in different combinations to remediate real contaminated soil. The purposefulness of application washing sequences, especially with saponin and rhamnolipids, for effective soil treatment was indicated. Further investigations on sequential washing with biosurfactants should be extended to other metal(oid)s, alone or co-occurring with organic pollutants in real contaminated soils. A holistic assessment of the quality of remediated soil and the stability of retained biosurfactants is needed.

## Figures and Tables

**Table 2:** Selected characteristics of aqueous solution of biosurfactants (mean ± SD, *n* = 3).

Characteristic	Biosurfactant		
	Saponin	Tannic Acid	Rhamnolipids
	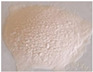	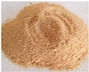	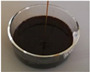
Chemical form	glycoside	polyphenol	glycolipid
Origin	plant	plant	microbial
Concentration, g TOC/L	13.6 ± 0.05	13.0 ± 0.4	13.3 ± 0.1
Density, g/mL	1.098 ± 0.003	1.085 ± 0.004	1.076 ± 0.003
pH	5.43 ± 0.11	4.38 ± 0.08	6.37 ± 0.13
Electrical conductivity (EC), mS/cm	31.3 ± 0.2	1.7 ± 0.0	36.6 ± 0.2
Surface tension, mN/m	43.0 ± 2.6	46.4 ± 1.8	23.6 ± 1.1

**Table 3:** Sequences of biosurfactant solutions for triple soil washing of smelter contaminated soil.

		Sequential Soil Washing		
No. of Sequence	No. of Biosurfactant Types in Sequence	Saponin (S) Variant	Tannic Acid (T) Variant	Rhamnolipids (R) Variant
1	1	S-S-S	T-T-T	R-R-R
2	2	S-S-T	T-T-S	R-R-S
3		S-T-S	T-S-T	R-S-R
4		S-T-T	T-S-S	R-S-S
5		S-S-R	T-T-R	R-R-T
6		S-R-S	T-R-T	R-T-R
7		S-R-R	T-R-R	R-T-T
8	3	S-T-R	T-S-R	R-S-T
9		S-R-T	T-R-S	R-T-S

## Data Availability

The data presented in this study are available on request from the corresponding author.
